# Editorial: Suicide in geriatric populations

**DOI:** 10.3389/fpsyt.2025.1670613

**Published:** 2025-10-07

**Authors:** Vincenzo De Luca, Xenia Gonda, Hanafi Damanhuri, Gary Cheung

**Affiliations:** ^1^ Department of Psychiatry, University of Toronto, Toronto, ON, Canada; ^2^ Department of Psychiatry and Psychotherapy, Semmelweis University, Budapest, Hungary; ^3^ Department of Clinical Psychology, Semmelweis University, Budapest, Hungary; ^4^ NAP3.0 Neuropsychopharmacology Research Group, Semmelweis University, Budapest, Hungary; ^5^ Department of Biochemistry, Faculty of Medicine Universiti Kebangsaan Malaysia (UKM), UKM Medical Center (UKMMC), Kuala Lumpur, Malaysia; ^6^ Department of Psychological Medicine, School of Medicine, Faculty of Medical and Health Sciences, The University of Auckland, Auckland, New Zealand

**Keywords:** suicide, aging, mental health, neurodegenerative disorders, euthanasia

Late-life suicide, particularly in men, is a significant public health issue in many parts of the world and it can have many devastating consequences for families, communities and health professionals (1). There is consensus that the spectrum of suicidality in late life (i.e., from death wish, suicidal ideation to completed suicide) is different from younger populations. Although there have been advances in identifying risk and protective factors for late-life suicide, there is still uncertainly about the precision of these predictions, particularly when applied at an individual level.

In older adults, epidemiological evidence showed ample differences in late-life suicide incidence across the globe and the role of sociocultural determinants in the development of suicidal behavior cannot be underestimated. Another important observation is the rising incidence of late-life suicide in some parts of the world over the last decades, despite the continuous progress made in screening and prevention. This paradox may be partially explained as increased susceptibility to suicide may be the consequence of marked cultural and environmental changes in the past two generations.

In this context, our Research Topic aimed to discuss the most important issues regarding suicide in this vulnerable population. To introduce this Research Topic, Sadek et al. provide a thorough narrative review of the current knowledge on late-life suicide. These authors provide an excellent overview of the current challenges, highlighting both risk and protective factors for suicide in the older population and summarizing the recent literature on fatal and non-fatal suicidal behavior, risk screening and suicide prevention.

The characterization of effective screening strategies for older people with prominent suicidal ideation and behavior represents one of the more relevant unmet needs of geriatric psychiatry. Nonetheless, investigations on new treatments aiming to reduce suicidal ideation are strictly intermingled with the characterization of the primary psychiatric diagnosis, and the elucidation of the biological underpinnings of both suicidal ideation and the primary disorder. These elements cannot be separated from each other, and their combined evaluation has been one of the main themes of this Research Topic. For example, Ramm et al. depict an intriguing topic article explaining that demoralization can be associated with suicidal ideation in older people.

In this Research Topic, we also have a contribution from Ethiopia where Siraj et al. present data from an African population emphasizing that there are few studies on suicide risks in Africa; therefore, the extent of late-life suicide in this continent may be underestimated. Siraj et al. found the risk factors for non-fatal late-life suicidal behavior in Ethiopia include being unmarried, chronic illness and elderly mistreatment. Using a large European sample, Conejero et al. confirm that previous suicide attempts are an important predictor of future suicidal acts and that health care cost can be different across different psychiatric diagnosis among older people, suggesting elevated suicide risk in older people may increase the burden for the health care system. Both observational studies from Africa and Europe suggest that parsing patient data considering specific age range is crucial to achieve more accurate prediction of suicide risk and similar approaches may pave the way to data-driven prediction in older populations to reduce suicide burden.

To complement the abovementioned quantitative studies, Hed et al. used a qualitative approach to examine the precipitants of late-life suicide attempts by interviewing older people in Sweden who have recently attempted suicide. They highlight that age-related losses may be exacerbated by negative interactions with the health system.

The factors associated with completed suicide are discussed in the contribution by Chan et al., where all deaths by suicide in people aged 60+ treated by the mental health services of the province of Alberta in Canada, during the last 25 years were reviewed to confirm that late-life suicide risk is different between males and females.

Similarly, Escher et al. have attempted to delineate the distinctive features of death by suicide in Honduras during the last 14 years, showing a post-COVID-19 pandemic increase in incidence, which is contrary to the global trend.

However, studies on completed suicide often face methodological limitations including small sample size and low statistical power; therefore, in their topic contribution, Meda et al. examined a large European dataset from the Survey of Health, Ageing, and Retirement in Europe (SHARE) study using a machine learning strategy. They found that the most important variables in the prediction model for late-life suicide were length of illness prior to suicide, number of offspring still alive and frequency of contact with next of kin, proposing that the risk for late-life suicide can be attributed to the construct of social connectedness.

The same SHARE prospective dataset was also analyzed in another remarkable Research Topic contribution by Zong and Wang, applying an XGBoost model to predict suicidal ideation among individuals over the age of 50. They found that childhood socioeconomic status is a specific predictor in this sample.

Regarding the neurobiological mechanisms of suicidal ideation and behavior in older people, Sessa et al. reviewed the current findings on monoamine systems and the hypothalamus-pituitary-adrenal axis in late-life suicide focusing on genetic and biomarker studies. Based on previous experimental studies, they suggest that the neurobiology of late-life suicide may stem from neurotransmitter imbalance and neuro-inflammation.

The theory of neurotransmitter imbalance related to suicidal ideation and behavior in older people is very relevant since antidepressants alleviate suicidal ideation in this population, whereas they can induce suicidal ideation in children and adolescents likely through increasing serotonin release and post-synaptic neuron activation. An age dependent mechanisms, due to multiple factors (i.e., reduced serotonin transporter expression as a consequence of age specific gene expression, or biological aging) may lead to the differential age effect of antidepressant on suicidal ideation (2–4).

The review by Sessa et al. addresses another important biological factor associated with late-life suicidal behavior such as neurodegeneration. Indeed, the relationship between cognitive impairment and late-life suicidal behavior has not been widely studied. Depression can worsen cognitive functioning in older adults, particularly in those who have a pre-existing neurodegenerative disorder. The traditional models of late-life suicide may have limitations when explaining suicide ideation in older people who have cognitive impairment. There could be a role for cognitive interventions such as cognitive stimulation, cognitive training and cognitive rehabilitation for older people with cognitive impairment who are at risk of suicide. However, the relationship between the severity of cognitive impairment and suicide risk is still unclear in existing literature.

Further research is urgently needed to explore specific models and neurobiological markers of suicidal ideation in older people, which may support the development of targeted therapies.

Sessa et al. also highlight the risk for suicide may change across the lifespan depending on multiple factors such as adverse life events and these factors can be different in the older population. For instance, older people are exposed to emergent medical issues and physical limitations, causing worsening of suicidal risk and making more difficult to predict suicidal behavior in this age group.

The issue of suicide prevention has been addressed in multiple articles. Pereira et al. describe the effect of a campaign in Brazil called Setembro Amarelo aimed to disseminate information related to suicide prevention. They found no difference in suicide rates in the 60+ population over a 12-years period. However, this study did not investigate the effect of the campaign on non-fatal suicidal behavior or other related outcomes such as hospital admissions related to suicidal behavior and ideation. This observation has a place into the current debate on understanding the effectiveness of culturally tailored prevention programs on late-life suicide.

Although the recommendation of guidelines for late-life suicide prevention programs is beyond the scope of this Research Topic, more focused efforts should be considered when designing suicide prevention program for this population, particularly for older men. This recommendation should be specifically applied to older people presenting with specific risks for suicidal behavior and medical burden, in contrast to younger people. Furthermore, this contribution strongly encourages systematic evaluation of suicide prevention programs across different countries.

Regarding medical assistance in dying (MAiD) that was one of the sub-themes of this Research Topic, the contribution of Castelli Dransart and Scozzari illustrates the results of a qualitative study about assisted suicide in old people living in long-term care facilities. Their results raised many questions about the most appropriate ethical, professional and organizational way to respond to the requests for suicide assistance and provide specific support to older people, families, and staff.

In summary, this Research Topic supports the distinction in suicidality between the older and younger populations; however, it also enlightens one of the limitations of current suicide prevention efforts as most of relevant strategies for preventing suicidal ideation and behavior are not tailored for age, gender and culture that are notoriously important factors in determining effectiveness.
